# Effects of species interactions on the potential for evolution at species' range limits

**DOI:** 10.1098/rstb.2021.0020

**Published:** 2022-04-11

**Authors:** Jake M. Alexander, Daniel Z. Atwater, Robert I. Colautti, Anna L. Hargreaves

**Affiliations:** ^1^ Institute of Integrative Biology, ETH Zurich, Universitätsstrasse 16, 8092 Zurich, Switzerland; ^2^ Biology Department, Earlham College, 801 National Rd. W, Richmond, IN 47374, USA; ^3^ Biology Department, Queen's University, 116 Barrie, St. Kingston, ON, Canada, K7 L 3N6; ^4^ Department of Biology, McGill University, 1205 Dr Penfield Av, Montreal, QC, Canada H3A 1B1

**Keywords:** range limits, trade-offs, biotic interactions, ecological release, niche expansion, local adaptation

## Abstract

Species’ ranges are limited by both ecological and evolutionary constraints. While there is a growing appreciation that ecological constraints include interactions among species, like competition, we know relatively little about how interactions contribute to evolutionary constraints at species' niche and range limits. Building on concepts from community ecology and evolutionary biology, we review how biotic interactions can influence adaptation at range limits by impeding the demographic conditions that facilitate evolution (which we term a ‘demographic pathway to adaptation’), and/or by imposing evolutionary trade-offs with the abiotic environment (a ‘trade-offs pathway’). While theory for the former is well-developed, theory for the trade-offs pathway is not, and empirical evidence is scarce for both. Therefore, we develop a model to illustrate how fitness trade-offs along biotic and abiotic gradients could affect the potential for range expansion and niche evolution following ecological release. The model shows that which genotypes are favoured at species' range edges can depend strongly on the biotic context and the nature of fitness trade-offs. Experiments that characterize trade-offs and properly account for biotic context are needed to predict which species will expand their niche or range in response to environmental change.

This article is part of the theme issue ‘Species’ ranges in the face of changing environments (Part II)’.

## 1. Introduction

Understanding why species have limited distributions along geographical gradients remains a fundamental challenge for ecologists and evolutionary biologists alike. Many range limits occur across continuous environmental gradients, where the geographical range can be thought of as the spatial manifestation of the species' niche (albeit sometimes heavily modified by dispersal [1]). Many such range limits seem to have remained stable geographically or to have tracked a specific environmental niche for thousands of generations [2,3]. From an evolutionary perspective, such range limits represent a failure of adaptive evolution to expand the niche, and so provide an opportunity to study the processes that constrain adaptation [4]. Evolutionary models of range limits typically consider a single species occupying an abiotic gradient [5–8]. In real ecosystems, however, species are never alone. Niche theory predicts that species are often excluded from some otherwise habitable areas by interactions with other species [9], and empirical approaches confirm that interactions can greatly modify species' distributions along abiotic gradients [10,11] and frequently contribute to species' range limits [12,13]. Unravelling the adaptive constraints that limit species' ranges in the wild, therefore, requires a framework that incorporates species interactions [14,15].

**Figure 1.**
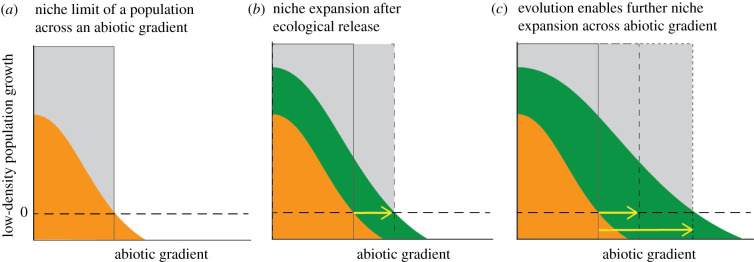
Conceptual illustration of how species interactions can limit evolution at a range edge. (*a*) A species’ range (grey box) occurs along the portion of an environmental gradient where the species has a positive growth rate (i.e. above the dashed reference line) at low intra-specific density but in the presence of other species. The range edge (black vertical line) occurs where the growth rate of a (potentially locally adapted) range-edge population (orange) falls below 0. (*b*) Ecological release following the loss or reduction of an antagonist (e.g. a competing species) increases population growth (green), enabling the species to expand its niche/geographical range ecologically without evolution occurring (yellow arrow). (*c*) In the longer term, ecological release allows the population to maintain larger population sizes, experience new portions of the abiotic gradient and experience a weakening of any adaptive trade-offs between the antagonist and the abiotic environment, enabling evolution and further range expansion.

Understanding how biotic interactions influence adaptation at range margins will not only shed light on how stable range limits are formed but also on how species' ranges change as environments change. For instance, the arrival of new antagonists (such as range-expanding species, invasive species, biocontrol agents) might shrink the breadth of environments within which a species is able to persist [16] or to which it is able to adapt [17]. Conversely, if populations lose antagonists (e.g. when introduced to a new range [18] or expanding into new regions in response to climate change [19]), escape from antagonistic biotic interactions will result in immediate ‘ecological release’ via increased population growth rate, potentially enabling persistence slightly further along the abiotic gradient (figure 1). This purely ecological effect could then be followed by ‘evolutionary release’ as species adapt to these new environmental conditions (figure 1).

There are multiple pathways along which altered species interactions could facilitate ‘evolutionary release’, as there are multiple ways in which species interactions can impede evolution. First, since species interactions influence demography, they could impede evolution by degrading the demographic conditions that facilitate adaptation [20]. For example, at range edges strong predation [21] could reduce population growth rates, competition could reduce the number of reproductive adults [22], or lack of pollinators could reduce outcross mating and, therefore, recombination [23]. Second, as biotic interactions themselves impose selection (e.g. for anti-herbivore/predator defences), they could stymie adaptive evolution by creating evolutionary trade-offs with the abiotic environment [1,24].

Several lines of evidence suggest that biotic interactions may commonly influence evolution at range edges in nature. Field studies that experimentally reduce the effect of negative interactions confirm that interactions often constrain fitness and that ecological release (increased performance when negative interactions are removed) is common [25]. Artificial selection experiments [26] and studies quantifying selection in nature [27,28] confirm that biotic interactions can often be important agents of selection (although this does not always translate into local adaptation [25]). A rich body of experiments shows that evolutionary adaptations to mitigate the fitness cost of a biotic interaction (e.g. herbivory) can incur a cost in performance (e.g. reduced growth) when the antagonist species is absent [29,30]. Finally, both intuition [31,32] and experiments [33,34] show that the intensity of interactions like competition and predation can vary predictably along environmental gradients, and species interactions commonly influence species' range limits [12]. Thus, we have robust evidence that biotic interactions can affect fitness, can impose selection and fitness trade-offs and are often correlated with abiotic gradients, such that interactions could frequently contribute to demographic and genetic constraints on evolution at range edges.

Here, our goal is to gain insight into how biotic interactions affect the potential for evolution at species' range limits, to better understand when ecological release from interactions would enable evolutionary expansion of a species' range. First, we use the classic Lotka–Volterra model of competition to clarify pathways by which biotic interactions could influence population growth and adaptation at range limits. Second, we summarize the modelling and empirical literature on how species interactions affect adaptation at range limits, to assess support for these pathways. Third, we combine the ecological Lotka–Volterra model with a fitness model to explore the conditions under which trade-offs involving biotic interactions could constrain adaptation and, therefore, species' ranges. We end by considering the basic and applied implications of our findings for the evolution of species' range limits.

## 2. How can biotic interactions affect adaptation at range edges?

One route to reconciling ecological and evolutionary perspectives on range limits is by recognizing that both species interactions and adaptive evolution influence range limits via population growth rate [20]. Specifically, negative species interactions can suppress population growth, creating or sharpening a range limit along an environmental gradient [14], whereas adaptation can increase population growth, expanding the species' distribution along the same gradient [15,35]. Consider a classic Lotka–Volterra model of competition, which predicts the abundance of a focal species (*N_i_*) in a given environment (for example, at a range edge), with an interspecific competitor (*N_j_*):

2.1 $$\frac{1}{N_{i}}\frac{dN_{i}}{dt}=r-\;\alpha N_{i}-\beta N_{j}.$$

In this model, *per capita* population growth of the focal species $\left(1/N_{i})(dN_{i}/dt\right)$ depends on the environment-dependent intrinsic growth rate (*r*) of the focal species, and the abundance and *per capita* effects of conspecific (*α*) and heterospecific (*β*) neighbours. Note that this two-species model may be expanded to a community of multiple interacting species. A species can colonize and persist in habitats where it has a positive population growth rate at low density (i.e. when *αN_i_* —the effect of conspecifics on population size—is negligible). As seen in equation (2.1), the low-density growth rate depends on the species' intrinsic growth rate (which may vary across an abiotic gradient) and on the abundance (*N_j_*) and impact (*β*) of antagonists.

Intuitively, if the strength of interspecific interactions (*βN_j_*) is relaxed, for example if an antagonist is lost, the low-density population growth rate of the focal species in that location will increase (‘ecological release’; figure 1*b*). If this happens across a species' range edge along a continuous environmental gradient, increased absolute fitness would mean that environments just beyond the current range would change from uninhabitable to habitable (i.e. low-density growth rate changing from less than or equal to 0 to greater than 0). Assuming dispersal was possible, ecological release should, therefore, enable the species to expand its range (figure 1*b*).

Ecological release from the negative effects of antagonistic biotic interactions (or a gain of positive interactions) should almost always increase demographic success and thus enable an ecological range expansion, but it could also facilitate additional evolutionary niche (and therefore range) expansion (figure 1*c*). One pathway to evolutionary range expansion is via demography (which we term a ‘demographic pathway to adaptation’), operating through effects of ecological release on population dynamics. Assuming ecological release enables a range-edge population to reach a higher carrying capacity via reduced interspecific density dependence, the population will reap the evolutionary benefits of larger population size. Larger populations at or near the range edge will be less prone to random demographic fluctuations and genetic bottlenecks, genetic drift eroding genetic diversity and asymmetric gene flow from central populations swamping beneficial adaptations [36–38] (though the extent to which gene swamping happens in nature is unclear [39]). Larger effective population sizes will increase the probability of beneficial *de novo* mutations and character combinations via greater genetic diversity and recombination; these effects combined with reduced genetic drift will increase the efficiency of selection [4,5,40].

A second pathway through which ecological release might facilitate evolution is by altering evolutionary trade-offs (i.e. a ‘trade-offs pathway to adaptation’). A range-edge population can face many, potentially conflicting, selection pressures. For example, there could be selection by environmental factors acting on its intrinsic growth rate (*r*), and selection by interspecific interactions that reduce fitness and population size via *β*. If underlying genetic or physiological constraints cause growth rates (*r*) to decrease as the effects of antagonists (*β*) decrease (for example, if investment in anti-herbivore defence, reducing *β*, incurs lower intrinsic growth), adaptation to an abiotic gradient will be constrained in the presence of those interactions, and adaptation to interactions will be constrained by the abiotic gradient. There can also be trade-offs in traits that affect a species' susceptibility to different interactions, as different biotic interactions can generate opposing selection pressures [41,42]. In both cases, loss or reduction of an enemy should reduce the trade-off. This in turn should allow more effective selection by other features of the environment, be they abiotic gradients or other interactions, enabling niche (and subsequently range) expansion.

A third pathway to evolutionary range expansion (or contraction), which we do not consider in detail here, is via dispersal. Interactions can affect dispersal directly (e.g. when animals disperse seeds or parasites) or indirectly (e.g. herbivores can reduce plant height, which reduces wind-dispersal of seeds). Dispersal strongly influences the evolutionary ecology of range edges by affecting gene flow within the range and colonization beyond it [43]. However, the effects of dispersal are complex, because increased gene flow to edge populations can both inhibit and aid adaptation [44,45] and because dispersal itself evolves, feeding back on range dynamics and evolution [46]. Here we greatly simplify things by ignoring dispersal, focusing on how interactions affect the potential for evolution via demography and adaptive trade-offs.

## 3. What do we know about how biotic interactions affect evolution at range edges?

### (a) Theory

Evolutionary biologists have long explored why species don't continuously expand their range via adaptation in edge populations. Early models examined range expansion of a single species along an environmental gradient (usually assumed to be abiotic), with adaptation to marginal habitat impeded when gene flow is too high (i.e. outbreeding depression or gene swamping [7]) or too low [45]. Some subsequent models incorporated biotic interactions that limit adaptation. Case & Taper [20] extended the classic single-species model [7] to include Lotka–Volterra competition. They showed that adding interspecific competition can generate range limits along shallower environmental gradients, by lowering population density and thus exacerbating the negative effects of maladaptive gene flow from the range centre [20]. Subsequent extensions added further nuance to the conditions under which competition and predation limit evolution and ranges along gradients with maladaptive gene flow [14,17,47]. Price & Kirkpatrick [15] showed how stable range limits can arise in the absence of gene flow when a competitor prevents the focal species from evolving to use a new resource along the gradient. Similar conclusions are reached in models revealing how competitors can stymie adaptation to changing environmental conditions via niche pre-emption in multi-species communities [48,49]. Generally, these models demonstrate how biotic interactions can create or exacerbate evolutionary limits on adaptation imposed by low population size. Nonetheless, while empirical studies show that adaptation to abiotic factors can affect the outcome of species interactions [50] and that populations can adapt to both abiotic and biotic factors [25], theory has so far not, to our knowledge, explored the conditions under which biotic interactions might limit ranges by generating adaptive trade-offs.

### (b) Empirical evidence

Empirical studies of whether biotic interactions affect evolution at range edges have lagged behind models, but there are some elegant examples supporting their potential effects. In a microcosm experiment of evolving archaea species, range expansion along a temperature gradient could be thwarted by the presence of a well-adapted competitor, as the competitor suppressed the abundance of the focal species [51]. This is consistent with a demographic pathway to adaptation (i.e. higher effective population size (*N*_e_) in the absence of competitors) and the models reviewed above. In a study of vernal pools, where annual plants have species-specific depth ranges, Emery & Ackerly [52] combined transplant and competitor-removal experiments to assess a focal species' adaptive potential. They showed that competitor removal enabled ecological release, allowing the species to occupy depths outside its normal range. Competitor removal also exposed genetic variation at the niche edge that was otherwise masked [52], though it is unclear whether this reflects the demographic pathway (e.g. higher *N*_e_) or trade-off pathway (i.e. variation no longer constrained by interactions). Either way, the fact that competitor removal increased expressed variation implies that evolution could expand the species’ niche and range limits in the absence, but not the presence, of competitors.

While evidence exists that trade-offs between traits under selection might contribute to setting range limits [53], there are few specific investigations into whether interspecific interactions are involved in these trade-offs. Trade-offs can arise when the abiotic and biotic environments impose antagonistic selection on a common trait. For example, Olsen *et al*. [54] created experimental crosses between high- and low-elevation populations of the small perennial plant *Boechera stricta* to expose genetic variation in trait combinations, then transplanted crosses across the species' low-elevation range limit. Both drought and herbivores imposed strong selection beyond the range, but did so antagonistically, resulting in a genetic trade-off that would prevent evolutionary niche and range expansion [54]. In another example, the distribution of the Trinidadian guppy *Poecilia reticulata* appears to be confined to freshwater by competition with a closely related competitor, which inhabits brackish water [55]. However, there is evidence that this ecological constraint is reinforced by an evolutionary constraint on adaption to brackish water, driven by a genetic trade-off between competitive ability and salinity tolerance [55]. In general, some traits, like phenology [56], may frequently influence both species interactions and adaptation to the abiotic environment, creating opportunities for trade-offs to constrain adaptation at range limits. Non-native species might offer some insight regarding the commonness of such trade-offs. This is because field studies suggest that specialist natural enemies are frequently lost upon introduction to new regions, selection is often relaxed in non-native relative to native species [57], and niche expansions in non-native regions occur frequently [58]. Nonetheless, we aren't aware of studies that have definitively linked niche expansion to a weakening of adaptive trade-offs involving biotic interactions.

## 4. Model of evolutionary trade-offs along biotic and abiotic gradients

As described in §3a, previous theoretical studies of biotic interactions at range edges have focused primarily on the ‘demographic pathway’ to adaptation, by linking ecological models of population growth to evolutionary models that allow genetic variation in the form of a shift in the optimum environment along an (unspecified) environmental gradient. However, multiple environmental variables simultaneously affect species' ranges (e.g. climate, soil quality or predator density). Further, because of genetic or physiological trade-offs, responses to one environmental variable might affect responses to other variables. To further explore the potential of the ‘trade-offs’ pathway to affect range limits, we next examine a complementary approach in which the environment consists of a biotic and an abiotic variable and a species' fitness is governed by trade-offs in response to biotic and abiotic stress. This type of model uses reaction norms that are agnostic to the genetic and mechanistic basis of trade-offs, which may include physiological or developmental constraints on trait evolution, or antagonistic selection on the same set of traits or genetic loci (see discussion in [18]). Furthermore, the model makes no assumptions about population size, and is, therefore, independent of any effects of ecological release on adaptation that operate via the first, demographic pathway. While not a formal evolutionary model, our approach allows us to illustrate the potential evolutionary consequences of selection for genotypes differing in a given trade-off as environments change. We note that this model is applicable to different modes of reproduction and inheritance, but its predictions are most intuitive for clonally evolving species with asexual reproduction and high homozygosity.

We describe a simple modification of the classic Lotka–Volterra model. Like previous models, we assume the growth rate of a population (or genotype) follows a Gaussian function along an environmental gradient, with peak fitness in the range core, declining to low fitness at and beyond the range edge (figure 1*a*). In other words, the species is at its environmental optimum in the range core, where its low-density growth rate is maximized (*λ*_max_). This is a simplification of the more general model of population growth in the absence of intraspecific competition, with populations occurring at locations *x* along an abiotic gradient, with an optimum environment *θ*_A_ located somewhere along that gradient [7].

### (a) Two dimensions of environmental variation

By contrast to previous models of species' range limits along an abiotic gradient, we here consider a defined biotic gradient, in which the position *y* on the biotic gradient represents the density of interspecific neighbours, with the optimum, *θ*_B_ = 0, corresponding to an optimum density of zero. Since our biotic gradient scales from 0 to 1, here we only consider antagonistic interactions. We allow the Gaussian fitness functions for both abiotic and biotic gradients to vary in their breadth (*V*_A_ and *V*_B_, respectively); a greater value of either indicates a greater niche breadth, meaning that fitness declines less severely as the species moves out of its environmental optimum. As detailed in the electronic supplementary material, an equation for fitness of an individual can be defined as a function of its biotic and abiotic environment:

4.1 $$W=\lambda_{\max}.\exp\left(-\frac{{\left(x-\theta_{A}\right)}^{2}}{2V_{A}}-\frac{{\left(y-\theta_{B}\right)}^{2}}{2V_{B}}\right)\;.$$

**Figure 2.**
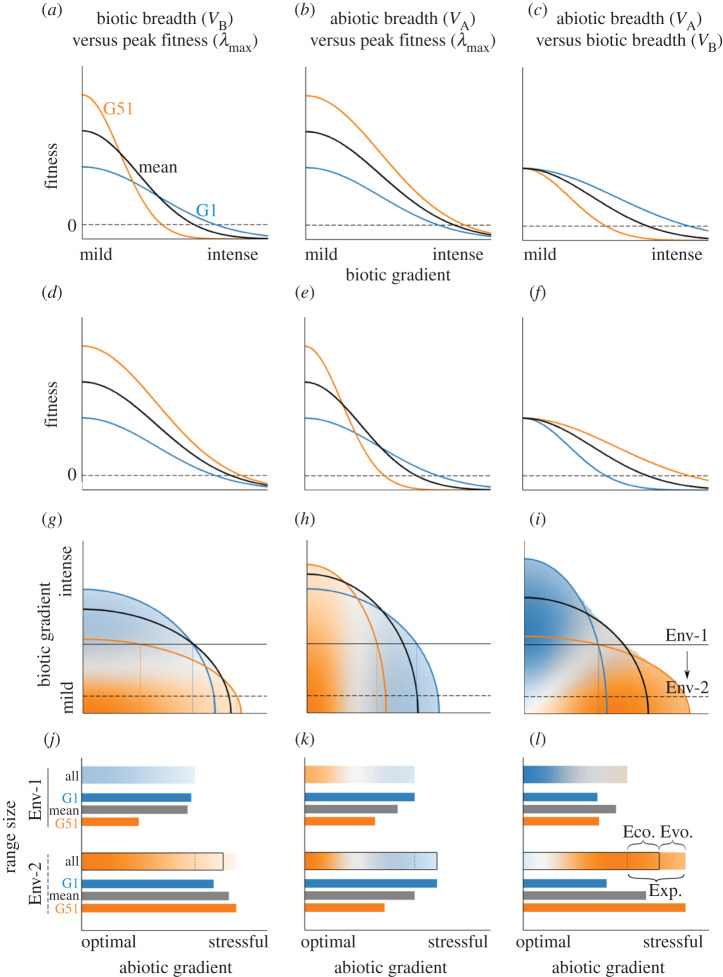
Fitness trade-offs affect responses to ecological release in a simple environment (species interactions are constant across an abiotic gradient). Each column corresponds to a trade-off between a pair of ‘traits’ (*a*–*f*) that determine the shape of the fitness function for 51 genotypes across biotic and abiotic gradients (*g*–*i*). Top row: the fitness of extreme genotypes, G1 and G51, and the mean genotype, vary along the biotic gradient from mild to intensely negative effects of interactions. Second row: the fitness of the same genotypes varies along the abiotic gradient, ranging from optimal to stressful. Third row: the identity of the most-fit genotype at each combination of abiotic (*x*-axis) and biotic (*y*-axis) conditions. Colour hue indicates genotype identity (blue: genotype G1, grey: mean genotype, orange: genotype G51), colour intensity indicates the strength of selection favouring that genotype, fading to white as fitness variation among genotypes approaches zero. Curved lines indicate the boundaries of the area of positive population growth for each genotype (*W* = 1; equation S4 in electronic supplementary material, Methods). Two hypothetical environmental axes are shown: Env-1 (benchmark axis, solid, in which biotic pressure is strong and constant along the abiotic gradient) and Env-2 (release from antagonists, dashed, in which biotic pressure is weak and constant along the abiotic gradient). The intercept between a genotype isocline and the environmental axis indicates the edge of the potential range (i.e. position along the abiotic gradient) for the three example genotypes in that environment. Bottom row: the possible ranges of the indicated genotype in environments 1 (Env-1; top bars) and 2 (Env-2; bottom bars). In each environment, the thicker, top bar is colour-coded with the identity of the fittest genotype at that position along the gradient. In Env- 2, this bar is surrounded by a square that represents the portion of the range that would be occupied if evolution did not occur. For reference, a dotted line shows the maximum range in Env-1. Brackets depict the total range expansion (Exp.), the portion of that expansion attributable to ecological effects of release from antagonists (Eco: the differences in ranges if there is no change in genotype at range margins), and the portion of that expansion attributable to evolutionary release via the ‘trade-offs pathway’ (Evo: the difference in ranges between the genotype favoured at the original range edge and genotype favoured at the expanded range edge). The thinner, solid-coloured bars show the total range of the genotypes G1 (blue) and G51 (orange), as well as the mean range of all genotypes (grey). Note that these are not necessarily the range edge genotypes.

Equation (4.1) allows us to explore how a series of genotypes fare across a set of environmental conditions, which we define as a specific combination of biotic and abiotic conditions that vary through space (‘environmental axes’, e.g. Env-1 and Env-2 in figure 2). For illustration in our model, we define a species' range as the locations along the fixed abiotic gradient where at least one genotype of the species has a positive population growth rate at low abundance (figure 2).

### (b) Incorporating evolutionary trade-offs

Whereas previous models have primarily considered genetic variation in one parameter (*θ*_A_), we consider the more general case of genetic variation in each of the five model terms *λ*_max_, *θ*_A_, *θ*_B_, *V*_A_ and *V*_B_. We treat the five model parameters as multivariate ‘traits’ of a genotype and introduce trade-offs by assuming that underlying genetic structure linearly affects more than one trait at a time. We create 51 genotypes (G1 to G51) that vary in *λ*_max_ and *V*_A_, with a trade-off represented by the negative genetic correlation among genotype means (e.g. as *λ*_max_ increases linearly, *V*_A_ decreases linearly). Modelling multiple genotypes at once is important because the relationship between trait values and fitness is curvilinear, which sometimes causes intermediate genotypes to be favoured. A full analysis of the ramifications of different covariance structures among model parameters is beyond the scope of this article. Instead, we provide three examples to illustrate how trade-offs can affect a species' range without invoking variation in dispersal, gene flow or population size (figure 2).

### (c) Potential evolution of range limits following ecological release: simple environmental gradients

We begin with two trade-off scenarios involving peak fitness and biotic or abiotic niche breadth. In the first example (figure 2, left column), biotic niche breadth (*V*_B_) trades off with peak fitness (*λ*_max_), such that the identity of the most-fit genotype varies along the biotic gradient (figure 2*a*) but not along the abiotic gradient (figure 2*d*). For example, plant genotypes that resist damage by insect herbivores (higher *V*_B_, blue line in figure 2*a*) have reduced fitness in the absence of herbivory (lower *λ*_max_) [29]. In the second scenario (figure 2, middle column), abiotic niche breadth (*V*_A_) trades off with peak fitness, such that the most-fit genotype varies only along the abiotic gradient (figure 2*e*). Conceptually, this is similar to plant species that can tolerate more stressful environments (higher *V*_A_) but don't perform as well as intolerant species when grown in favourable environments (lower *λ*_max_) [59]. Given these trade-offs, we can apply equation (4.1) to explore the relative fitness of genotypes in a variety of environments, beginning with a relatively simple case in which biotic interactions are independent of the abiotic gradient (Env-1 in figure 2*g*). When biotic niche breadth trades-off with peak fitness (figure 2 left column), the most-fit genotype is the same everywhere along the abiotic gradient (i.e. the gradient without the trade-off; figure 2*g*). Conversely, when abiotic niche breadth trades off with peak fitness (figure 2 middle column), the most fit genotype varies along the abiotic gradient (figure 2*h*).

In our model, potential range expansion occurs through both ecological release and evolutionary release—via the trade-off pathway—as biotic conditions change (e.g. moving from Env-1 to Env-2 in figure 2). However, the contribution of ecological and evolutionary effects depends on the nature of the trade-offs (i.e. trait covariance). In all scenarios, ecological release increases fitness (*W*) of all genotypes, increasing the potential range of each genotype (compare length of top versus bottom bars in figure 2 bottom row) and the species' overall range. However, the degree of release varies among genotypes (compare relative increase in length of blue versus orange bars in figure 2 bottom row), and the identity of the most-fit genotype at the range edge sometimes changes between environments, depending on the trade-off structure. Range expansion via the trade-off pathway occurs when (i) there is a shift in the favoured genotype at the range edge and (ii) the range of the new most-fit edge genotype expands the range of the whole species (e.g. range of orange bar in Env-2 > range of blue bar in Env-2; figure 2*j*). In other words, range expansion via the trade-off pathway occurs when the total range expands and the identity of the most-fit genotype on the range edge also changes. In the first trade-off scenario (*V*_B_ versus *λ*_max_), reducing negative biotic interactions changes the most-fit genotype from the highest niche-breadth (blue) to peak-fitness (orange) genotype along the entire abiotic gradient, but all genotypes have similar ranges in the new environment (figure 2*j*). Thus, evolution is predicted to have little potential contribution to range expansion relative to ecological release. In the second trade-off scenario (*V*_A_ versus *λ*_max_), range expansion after reducing antagonists is due entirely to ecological release because the identity of the most-fit genotype does not change anywhere along the abiotic gradient (colour gradient is at the same position for Env-1 and Env-2; figure 2*k*).

In a third trade-off scenario, abiotic niche breadth (*V*_A_) trades off with biotic niche breadth (*V*_B_; figure 2, right column). This might occur if traits that enable an organism to adjust to the presence of an antagonist (e.g. increased canopy growth in plants competing for light) constrain its abiotic niche (e.g. high shoot investment limits the ability to grow in stressful habitats) [59]. Reducing negative interactions again expands the potential range (increase in length of all-genotype bar in figure 2*l*) via both ecological release and the trade-off pathway, manifested by the change in the identity of the most-fit genotype throughout the range (compare colours of all-genotype bar in Env-1 versus Env-2; figure 2*l*). By contrast to the other two scenarios, the amount of both ecological and evolutionary release is greater, and the intensity of selection changes throughout the range (strongest in range centre in Env-1, weakest in range centre in Env-2). A key feature of this third trade-off scenario is that the variance in fitness among genotypes covaries with both the abiotic gradient and the biotic gradient (diagonal gradient shading in figure 2*i*). As a result, the relationship between the environmental axis, which describes covariance between environmental variables, and the fitness surface, which is determined by covariance between model parameters, strongly affects the relative contributions of ecological release and the trade-off pathway following release from antagonists.

### (d) Range expansion following ecological release: more complex environmental gradients

**Figure 3.**
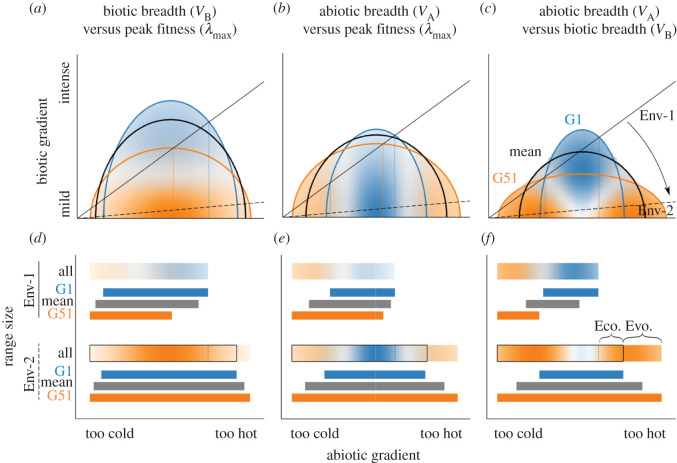
Trade-offs affect responses to ecological release in a complex environment (biotic and abiotic conditions covary). While fitness trade-offs are the same those as shown in figure 2, here the focal species' fitness along the abiotic gradient is maximized at intermediate values (the abiotic gradient is given as temperature but could be any abiotic variable). As in figure 2, two hypothetical environmental axes are shown, but here the environmental axes (Env-1, the benchmark axis, solid; Env-2, the ecological release axis, dashed) are sloped. This corresponds to a scenario in which biotic stress increases along the abiotic gradient, so that biotic release has the greatest effect where biotic stress is highest. The abiotic range limits are defined as the points where the isocline crosses the environmental axis. As a result, species distributions in Env-1 are skewed to the left, because moving right along the environmental axis means moving into increasingly intense biotic inhibition. Colour corresponds to genotype (see figure 2); in top panels and the all-genotype bar in bottom panels, colour hue indicates the most fit genotype and colour intensity indicates the intensity of selection; see figure 2 for full description of panels.

In real environments, the intensity of biotic interactions may covary with abiotic gradients, and species’ ranges can be limited by complex interactions between them. For example, the importance of plant competition can increase from abiotically stressful to fertile conditions [59,60], and predation can increase from high to low latitudes [33,34]. Further, species typically have an abiotic optimum along an abiotic gradient, such that fitness declines as abiotic conditions depart from that optimum in either direction (e.g. along a temperature gradient fitness might be limited by heat at one end and by cold at the other). We next explore such an environment, where fitness is maximized at the centre of an abiotic gradient and biotic interactions covary with the gradient (figure 3). This could be envisioned as a species occupying an elevational band along a mountain, where the lowlands are too hot and the highlands too cold, and where the intensity of competition increases from highlands to lowlands. In this model, the loss of antagonists ameliorates the biotic environment most strongly where interactions are most intense, resulting in a weaker environmental correlation (Env-1 versus Env-2 in figure 3).

We can explore the effects of the same genetic trade-offs depicted in figure 2 in this more complex and realistic environment. In all three trade-off scenarios, range expansion only occurs at the range edge that had the strongest biotic interactions (figure 3); thus, we focus our discussion below on the biologically intense end of the range. In the first environment (Env-1), biotic stress limits the range near the species' abiotic optimum, whereas in the more biotically benign environment (Env-2), the species can occupy the environment until it reaches abiotically marginal habitat. When the breadth of the biotic niche trades-off with peak fitness (*V*_B_ versus *λ*_max_; figure 3 left column), potential range expansion is driven primarily by ecological effects because all genotypes expand their range, though the dominant genotype shifts from G1 (blue; higher *V*_B_) to G51 (orange; higher *λ*_max_). These changes resemble the case of uncorrelated environments (compare figure 2*j* and figure 3*d*). Although the identity of the genotype with the highest fitness changes, little additional range expansion would be gained by evolution from blue to orange in Env-2 (compare bars in figure 3*d*). When *V*_A_ and *λ*_max_ trade off (figure 3 middle column), release from enemies enables a larger potential range expansion driven mainly by evolution (i.e. changing from the most-fit edge genotype in Env-1 (blue; higher *V*_A_) to the most-fit edge genotype in Env-2 (orange; higher *λ*_max_); figure 3*e*). When abiotic versus biotic niche breadth trade off (figure 3 right column), the trade-off pathway again contributes strongly to range expansion at the biotically intense end of the environment. When biotic limitation is strong, the ‘biotic-breadth’ (blue; higher *V*_B_) genotype is favoured, whereas when biotic limitation decreases, the ‘abiotic-breadth’ (orange; higher *V*_A_) genotype becomes favoured at both ends of the range, functioning as a ‘general-purpose genotype’ [61,62]. In all three scenarios, ecological release also intensifies selection, though where selection is strongest depends on the nature of the trade-off (compare location of most intense colour in ‘all genotype’ bars; figure 3*d*–*f*).

### (e) Summary of model insights

Our analysis of just three relatively simple trade-off models demonstrates how the loss of antagonists (ecological release) can have complex effects on the potential for range expansion and evolution. Predictions are highly contingent on both environmental and trait correlations, but some general trends emerge. (i) Ecological release is always followed by an expansion of the occupiable range, except in environments where enemy abundance is already low (e.g. at the biotically mild end of the abiotic gradient in figure 3). (ii) Ecological release can change which genotype is favoured at range edges, such that evolution via the trade-off pathway could contribute, sometimes substantially, to range expansion. (iii) Even when the identity of the favoured genotype changes at the range edge, evolution may contribute less to the total range expansion than ecological effects (figures 2 and 3, left column). (iv) Which genotypes are favoured at any location depends on covariance between the environmental axis and the fitness surface. (v) Ecological and evolutionary release might act synergistically to expand the potential range (figure 1), but the relative contribution of each depends on the nature of the traits under selection, their correlations with one another, and their correlations to underlying axes of environmental variation, not to mention whatever processes link selection to evolution and *r* to actual population growth. These complexities were apparent even disregarding other biotic processes known to contribute to evolution at range edges; future work could expand our relatively simple, illustrative models to more formal models that incorporate different trade-off structures (i.e. covariance matrices) and environmental axes, as well as dispersal, gene flow, drift, sexual reproduction and density-dependence (i.e. *α* and *β* in equation (2.1)). Finally, while we have discussed our model results mostly in terms of biotic release, other environmental changes can be similarly modelled. For example, many native species gain new antagonists following range expansions and introductions of non-native species, which could result in a broad array of potential range dynamics (i.e. shifting from Env-2 to Env-1 in figures 2 and 3). More generally, trade-off models like ours can help to explore ecological and evolutionary responses to climate change and other forms of global change.

## 5. Basic and applied implications for the evolution of species' range limits

The most important take-home message from our model is that the biotic context for adaptation along abiotic gradients cannot be ignored if we hope to make evolutionary predictions for wild species and ecosystems. Our model shows that selection along environmental gradients can be profoundly influenced by fitness constraints imposed by biotic interactions and genetic trade-offs in adaptation to the abiotic and biotic environments. This has implications for empirical approaches to studying adaptation at range limits and for managing species that are expanding their ranges or responding to environmental changes at their range limits.

Our study highlights the need for studies that simultaneously consider impacts of both abiotic and biotic selection pressures at range edges, inferred from laboratory conditions and conducted in nature. The most robust and nuanced understanding of fitness constraints and adaptive potential at real range edges comes from manipulative field experiments, for example transplanting species beyond their current range to quantify fitness limitation, or among populations to quantify local adaptation and plasticity in edge populations (e.g. [52,55]). However, these experiments often use unrealistic genotypes (e.g. range centre populations, hybrid lines) or alter the biotic context (e.g. weeding all plots). While these modifications can help answer specific questions, they can also strongly influence resulting estimates of the niche, local adaptation and adaptive potential [25,63]. As our model shows, altering the biotic context can alter the strength and direction of selection on traits caused by the abiotic environment. To understand the ecological and evolutionary processes limiting species' distributions in the wild, we need to measure them in the fully complex environmental context.

Understanding the evolution of species' ranges is increasingly relevant to conservation. As native and introduced species respond idiosyncratically to global change, old interactions may be lost and novel ones produced [64]. Adaptation to novel interactions at range edges can facilitate range expansion, as seen in the rapid northward expansion of a butterfly following adaptation to a new host plant [65,66]. For slow-moving species, adaptation may be required simply to maintain existing populations in the face of novel competitors [67]. Therefore, estimating the importance of other species as agents of selection at range edges, and the ability of focal species to respond appropriately, might help explain when range-edge populations will persist, expand or collapse as environments change. There is abundant evidence for niche expansion during invasion [58,68], but demonstrations of niche evolution in the native range on contemporary timescales are scarce [69,70]. Future work might expand our modelling approach to explore the configuration of biotic and abiotic conditions that make evolutionary niche expansion most likely, and hence invasions least predictable. In sum, our study highlights that adaptation to the abiotic environment can depend strongly on the biotic context and emphasizes the urgent need for empirical studies and distribution models that tackle the eco-evolutionary complexity of species' range limits.

## Data accessibility

Code has been uploaded as electronic supplementary material [71].
